# A novel combined quadrivalent self-amplifying mRNA-LNP vaccine provokes protective immunity against acute and chronic toxoplasmosis in mice

**DOI:** 10.1186/s40249-025-01332-6

**Published:** 2025-06-23

**Authors:** Qinli Wu, Zhongao Zhang, Hongkun Chu, Bing Xia, Weiqi Li, Jianzu Ding, Haojie Ding, Bin Zheng, Meng Gao, Youru Wang, Eman E. El Shanawany, Feng Tan, Huayue Ye, Xunhui Zhuo, Shaohong Lu

**Affiliations:** 1 School of Basic Medical Sciences and Forensic Medicine, Hangzhou Medical College, Hangzhou, 310013 China; 2 Engineering Research Center of Novel Vaccine of Zhejiang Province, Hangzhou Medical College, Hangzhou, China; 3 GuoTai (Taizhou) Center of Technology Innovation for Veterinary Biologicals, Taizhou, China; 4 Department of Parasitology and Animal Diseases, Veterinary Research Division, National Research Centre, Cairo, Egypt; 5 Department of Parasitology, School of Basic Medical Sciences, Wenzhou Medical University, Wenzhou, China

**Keywords:** *Toxoplasma gondii*, mRNA vaccine, Self-amplifying, Immune protection, Oocyst

## Abstract

**Background:**

*Toxoplasma gondii*, an intracellular parasitic protozoan, which infects almost all warm-blooded animals, including humans, causes toxoplasmosis. However, we lack effective drugs and vaccines to control toxoplasmosis, representing a clinical challenge. Therefore, safe and effective vaccines are urgently needed. In this study, a self-replicating mRNA vaccine comprising four *T. gondii* antigens: ROP18, TGME49_237490, TGME49_268230, and MIC13, named 4x-mRNA-LNP (lipid nanoparticle), was developed, and its protective efficacy was evaluated in mice.

**Methods:**

The expression of this vaccine in eukaryotic Human embryonic kidney 293 T (HEK-293 T) cells and mouse myoblast (C2C12) cells were analyzed, followed by enzyme-linked immunosorbent assay (ELISA) evaluation of the elicited humoral immune response. Subsequently, the vaccine-triggered immune responses in mice were detected, including antibody titers, T lymphocyte subsets, and cytokine levels. Finally, its immunoprotective effects were evaluated after challenging mice with *T. gondii* PRU oocysts or tachyzoites of different strains and analyzing the pathological changes, parasite loads, and mouse survival time. Western blotting and ELISA confirmed the successful eukaryotic expression and immunogenicity of 4x-mRNA, respectively. Statistical analyses, including the log-rank (Mantel–Cox) test, Student’s* t*-test, and one-way ANOVA, were performed using GraphPad Prism software.

**Results:**

Mice vaccinated with 4x-mRNA-LNP generated higher levels of IgG1 and IgG2a antibodies (*P* < 0.05) and cytokines (IL-2, IL-4, IL-10, IL-12, IFN-γ) (*P* < 0.05) compared with the control group. The high specific IgG titer was maintained for at least 10 weeks after the last vaccination. The proportion of CD3^+^CD4^+^ T cells and CD3^+^CD8^+^ T cells also increased significantly (*P* < 0.05), along with increased spleen cell proliferation in 4x-mRNA-LNP-vaccinated mice. Notably, limited pathological changes and < 10 fg of parasites/mg were found in the immunized mice tissues post-pathogen challenge. During observation for 30 days, 4x-mRNA-LNP-immunized mice survived significantly longer under challenge with lethal doses of RH, ME49, or WH6 tachyzoites (survival rates = 60%, 80%, and 60%, respectively). Following PRU oocyst challenge, vaccinated mice had notably decreased cyst burdens (72.5%, *P* < 0.05) compared with control mice.

**Conclusions:**

The 4x-mRNA-LNP vaccine triggered effective long-term antibody levels in mice, thus representing a promising candidate to further develop anti-toxoplasmosis vaccines.

**Graphical Abstract:**

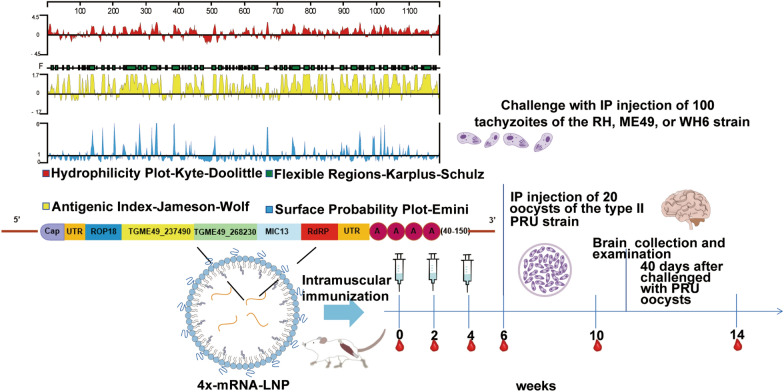

**Supplementary Information:**

The online version contains supplementary material available at 10.1186/s40249-025-01332-6.

## Background

*Toxoplasma gondii*, the causative agent of toxoplasmosis, is a zoonotic parasite with a worldwide distribution that presents a profound risk to public health [1]. It can infect almost all warm-blooded animals, including humans [2]. The global average seroprevalence of *T. gondii* is approximately 25.7%, with a particularly high rate of 61.4% being observed in Africa [3]. Generally, people with normal immunity usually have no obvious symptoms when infected by *T. gondii*. However, for immunodeficient individuals, such as organ transplant patients and patients with AIDS, toxoplasmosis is one of the common causes of death, often leading to toxoplasmic encephalitis and peritonitis [4]. In addition, *T*. *gondii* can infect the fetus through the placental barrier [5], thereby directly affecting the fetus and even leading to miscarriage, premature birth, or stillbirth, and posing a serious threat to eugenics. Therefore, it is vitally important to implement effective prevention and treatment methods against toxoplasmosis. Vaccination is considered an effective approach for disease control and prevention [6]. Over the past three decades or more, extensive experimental studies highlighting anti-*T*. *gondii* infection measures that aim to reduce or eliminate the formation of tissue cysts have been carried out, involving live attenuated vaccines [7], subunit vaccines, DNA vaccines, mRNA vaccines, and others. Currently, the only commercialized *T. gondii* vaccine, Toxovax^®^ [8], is a live attenuated vaccine developed from the attenuated S48 strain, which is primarily used in goat farming and carries a risk of virulence reversion. DNA vaccines can promote the specific expression of antigens encoded by the vaccine in host cells and trigger stronger humoral and cellular immunity than that of subunit vaccines. For example, *T*. *gondii* subunit vaccines containing recombinant granule antigen protein 2 (rGRA2) or rGRA5 provided partial protective effects to vaccinated BALB/c mice [9]. The DNA vaccine encoding the *Toxoplasma* GRA14 antigen could induce a strong specific immune response, and the average survival time of immunized BALB/c mice was significantly prolonged [10]. In contrast to DNA vaccines, mRNA vaccines work effectively once they pass through the cell membrane and enter the cytoplasm, while plasmid DNA needs to move into the nucleus for further function. Thus, mRNA vaccines could produce a stronger immune response with higher safety because the risk of the integration of foreign genetic material into the host chromosome has been eliminated. Chahal et al. [11] prepared a *Toxoplasma* mRNA vaccine simultaneously carrying sequences encoding GRA6, rhoptry protein 2 A (ROP2A), ROP18, surface antigen 1 (SAG1), SAG2A, and apical membrane antigen 1 (AMA1). Immunized mice developed a high level of immunity against the lethal dose of the *T. gondii* type II PRU strain (hereafter referred to PRU). In contrast to traditional mRNA vaccines that require cellular ribosomes for protein production, the self-replicating mRNA used in this study carries the sequence of RNA-dependent RNA polymerase (RdRp) [12], which could be used as a template to produce more cRNA, thereby significantly increasing the duration of mRNA in the cells and enhancing the immune response. The main vaccine candidate molecules for *T*. *gondii* focus on SAGs, ROPs, microneme proteins (MICs), and GRA proteins [13]. Among potential vaccine targets, ROP18 and MIC13 stand out for their critical roles in parasite pathogenesis. ROP18 facilitates intracellular survival by phosphorylating host cell proteins to evade immune detection, while MIC13 mediates crucial adhesion and invasion processes. Notably, MIC13 shows significant upregulation during chronic infection and bradyzoite differentiation [14], and both antigens have proven to be effective in DNA vaccine formulations against acute and chronic toxoplasmosis [15, 16]. To expand our target repertoire, comprehensive transcriptomic analysis of the sexual reproductive stage identified two additional key antigens: the centrin family protein TGME49_237490 and oocyst wall protein (OWP) ortholog TGME49_268230, which show marked upregulation during oocyst development [17]. Their stage-specific expression patterns strongly suggest that they have essential roles in sexual reproduction, making them ideal candidates for disrupting parasite transmission. Building upon these findings, we developed an innovative multi-antigen vaccine platform using self-replicating mRNA technology. In addition, many studies have shown that vaccines targeting a single antigen or a small combination of antigens provide limited anti-*T*. *gondii* effects, while multi-antigen combined immunization can induce stronger immune responses [18]. Thus, the multi-antigen targeted self-replicating mRNA vaccine designed in this study could have a significant protective effect against toxoplasmosis.

In this study, a self-replicating mRNA vaccine containing four antigens that are all key proteins in different life stages of *T*. *gondii*: ROP18, TGME49_237490, TGME49_268230, and MIC13 (abbreviated as 4x-mRNA), was designed to combat acute and chronic toxoplasmosis, and even the infection caused by oocysts from the sexual reproductive stage. The immune protective effects of 4x-mRNA were further investigated in a mouse model. In addition, the potential mechanisms and prospects of the 4x-mRNA vaccine were discussed with reference to the immunogenicity and humoral and cellular immune responses induced by vaccination.

## Methods

### Mice

Six- to eight-week-old BALB/c and Institute of Cancer Research (ICR) mice were purchased from the Center of Laboratory Animals of Hangzhou Medical College (Hangzhou, China). All mice used in the experiments were handled humanely according to the Animal Ethics Procedures and Guidelines of the People’s Republic of China. The experimental protocols were approved by the Animal Care and Use Committee of Hangzhou Medical College (approval number: 2023-018).

### Parasite and cell culture

Tachyzoites of the ME49, WH6, and RH strains of *T. gondii* were passaged in ICR mice. Human embryonic kidney 293 T (HEK-293 T) cells and mouse myoblast (C2C12) cells were cultured in high-glucose Dulbecco’s Modified Eagle Medium (DMEM; Gibco at Thermo Fisher Scientific, Waltham, MA, USA) supplemented with 10% Fetal Bovine Serum (FBS; Gibco) at 37 °C, and 5% CO_2_. In all experiments, freshly harvested tachyzoites from mice ascites were filtered through a 5-µm polycarbonate membrane to remove host cell debris. The PRU oocysts used in this study were kindly provided by Professor SY Huang (Yangzhou University, Jiangsu Province, China).

### Design, construction, and 4x-mRNA encapsulation with lipid nanoparticles (LNPs)

Four genes or epitopes of *T*. *gondii* from various life stages/periods, namely ROP18, TGME49_237490, TGME49_268230 and MIC13, were acquired through analysis at the Toxo database (ToxoDB, http://toxodb.org/toxo/). The core epitopes of ROP18 (amino acids 348–554), TGME49_237490 (amino acids 41–251), TGME49_268230 (amino acids 41–316), and MIC13 (amino acids 48–468) were selected and modified with a C-terminal his-tag. The complete coding sequence of the four His-tagged epitopes was then codon-optimized for expression in mice (the full sequences are provided in Supplementary material 1). The optimized sequence was then ligated to the DNA encoding the RdRp of Venezuelan equine encephalitis virus (VEEV), which is jointly formed by nonstructural proteins nsP1–nsP4. The resulting RdRp-4 × construct was cloned into a T-vector. The RdRp-4 × sequence was linearized using *Sal* I and *Bam*H I double enzyme digestion, and in vitro transcription was performed using a MEGAScript™ Kit (Thermo Fisher Scientific) as previously described [19]. The purified transcribed RNAs were capped with Cap1 (m7GpppXm) using the Vaccinia Capping System (NEB, Ipswich, MA, USA) and poly-A tail structures were added enzymatically according the manufacturer's instructions before encapsulation.

The LNPs were then produced by the ethanol dilution process at a molar ratio of 10 [1,2-distearoyl-sn-glycero-3-phosphocholine (DSPC, Sigma, St. Louis, MO, USA)]:48 [cholesterol (Sigma–Aldrich, St. Louis, MO, USA)]:2 {1,2-dimyristoyl-sn-glycero-3-phosphoethanolamine-N-[methoxy (polyethylene glycol)−2000] (ammonium salt) (PEG DMG 2000) (Avanti Polar Lipids, Alabaster, AL, USA)}:40 [1,2-dilinoleyloxy-3-dimethylaminopropane (DLinDMA)] as previously described [19]. The self-amplifying RNA (saRNA) vaccines in 100 mmol/L citrate buffer were encapsulated in LNPs via spontaneous vesicle formation [20], followed by buffer-exchange with phosphate buffered saline (PBS) to yield the final 4x-mRNA-LNP product. Additionally, the potential epitopes of the 4 × protein were predicted using DNASTAR software (Madison, WI, USA).

### mRNA/LNP expression in vitro

To evaluate the in vitro expression of 4x-mRNA-LNP, HEK-293 T cells (1 × 10^6^) were seeded in six-well plates and subsequently transfected with 4x-mRNA-LNP to assess mRNA translation. To further detect whether mRNA can be expressed by intramuscular injection, 4x-mRNA-LNP was transfected into C2C12 skeletal muscle cells. After 72 h of culture, the supernatant was discarded and 100 μl of Radioimmunoprecipitation assay (RIPA) buffer (Beyotime, Shanghai, China) was added to each well to collect cell lysates. Subsequently, the expression of the 4 × protein was detected using western blotting. Briefly, the lysates were subjected to sodium dodecyl sulfate–polyacrylamide gel electrophoresis (SDS-PAGE), and the proteins on the gel were then transferred to nitrocellulose (NC) membranes (Sigma–Aldrich). After being blocked with PBS solution containing 0.05% Tween 20 (PBST) and 5% bovine serum albumin (BSA) at room temperature (RT) for 2 h, the NC membranes were washed three times with PBST and incubated with anti-glyceraldehyde-3-phosphate dehydrogenase (GAPDH) primary antibodies (Abcam, Cambridge, UK, 1:4000) at RT for 2 h. After three washes with PBST, horseradish-peroxidase (HRP)-conjugated goat anti-Rabbit (Abcam, 1:5000) or HRP-conjugated anti-His-Tag (Proteintech Group Inc., Rosemont, IL, USA, 1:5000) were added as secondary antibodies and incubated at RT for 1 h. After three washes, the bands were visualized using an enhanced chemiluminescent chromogenic substrate (Beyotime).

### Immunization and* T. gondii* challenge

Mice were immunized intramuscularly three times with 15 μg of 4x-mRNA-LNP vaccine in 100 μl of PBS, an equivalent volume of PBS containing only LNPs, or an equivalent volume of pure PBS (*n* = 40 per group). Serum samples were collected from the tail vein of each mouse at two-week intervals (weeks 0, 2, 4, 6, 10, and 14) and stored at − 20 °C for subsequent analyses. Two weeks after the last immunization, mice were intraperitoneally injected with 1 × 10^2^ tachyzoites of the RH, ME49, or WH6 strain, respectively, and the survival times were recorded daily until 30 days post-infection (dpi). At the same time, mice were intraperitoneally injected with 20 PRU oocysts and euthanized at 40 dpi. The brains of the mice in each group were collected and ground into homogenized suspensions. Through microscopic observation, the number of cysts in each brain homogenate was counted to determine the cyst load in the brain.

### Preparation of the 4 × recombinant protein

To obtain the 4 × recombinant protein, we established a eukaryotic expression system by transfecting 293 T cells with 4x-mRNA-LNP, as previously described. Following transfection, the cells were lysed using RIPA buffer followed by protein purification through an Ni–NTA (his-tag) prepacked gravity column (Sangon Biotech, Shanghai, China). Purification was conducted using a stepwise imidazole gradient elution protocol (25, 50, 100, 200, and 500 mmol/L in urea-containing buffer). The collected elution fractions subsequently underwent SDS-PAGE separation and western blotting analysis, employing HRP-conjugated anti-His tag antibodies for protein detection.

### Measurement of antibody levels in mice

The levels of IgG antibodies, as well as the IgG1 and IgG2a subtype antibodies in mice were evaluated through an enzyme-linked immunosorbent assay (ELISA). Briefly, the 4 × protein was diluted to a concentration of 5 μg/ml using bicarbonate buffer (pH 9.6) and added to 96-well plates (100 μl per well) for overnight incubation. The coated plates were washed five times with PBST and then blocked with PBST containing 5% skimmed milk powder at 37 °C for 1 h. After five washes with PBST, 100 μl of serum samples diluted with PBST containing 5% skimmed milk powder (1:100) were added as primary antibodies to the wells and incubated at 37 °C for 1 h. The plates were washed five times and then 100 μl of diluted HRP-conjugated goat anti-mouse IgG (Abcam, 1:10000), IgG1 (Abcam, 1:5000), or IgG2a (Abcam, 1:5000) antibodies were added as secondary antibodies, and incubated at 37 °C for 1 h. After five washes, 100 μl of 3,3′,5,5′-tetramethylbenzidine (TMB) chromogen solution (Beyotime) was added and the plates were incubated at 37 °C for 15 min. Finally, 50 μl of 2 mol/L H₂SO₄ was added to terminate the reaction, and the absorbance value at 450 nm was read using an ELISA microplate reader (BioTek, Instruments Inc., Winooski, VT, USA).

### Flow cytometry analysis of T cell subsets

To analyze the T cell subsets, mice in each group were immunized as described above. Two weeks after the last immunization, 200 μl of whole blood was obtained from the mice and transferred into a sample tube. Red blood cell lysis buffer (Sigma) was added and the cells were lysed at RT for 2–3 min. The sample were centrifuged at 400 × *g* for 5 min and the supernatant was discarded to remove the red blood cells. Subsequently, 100 μl of PBS with 0.5% FBS was used to resuspend the peripheral blood mononuclear cells (PBMCs), followed by the addition of the blocking antibody and incubation in dark for 20 min. Then, fluorochrome-labelled monoclonal antibodies (mAbs), including FITC-labeled anti-CD3, PerCP-Cy5.5-labeled anti-CD4 and APC-Cy7-labeled anti-CD8, (BD Biosciences, Franklin Lakes, NJ, USA) were added for 30 min of incubation in the dark. The mixture was washed with 1 mL of FACS, followed by centrifugation at 400 × *g* for 5 min. The sample was resuspended in 500 μl of FACS. The T cells were analyzed using a flow cytometer (BD Biosciences) employing FlowJo software (BD Biosciences).

### Cytokine assay

To determine the level of cytokines, mouse splenocytes were harvested aseptically and made into a single-cell suspension (*n* = 5). In brief: The spleen was transferred to a 70 μm cell sieve (with the sieve placed in a 50 mL centrifuge tube) and slowly ground with the inner core of a 5 mL syringe while sterile FACS was slowly dripped during grinding. The collected cell suspension was then centrifuged at 400 × *g* for 5 min, and the red blood cells were lysed and removed as described above. Finally, the splenocytes were obtained and resuspended in DMEM. The total number of cells was counted using an improved Neubauer counting chamber (WATSON, San Diego, CA, USA), and splenocytes (1 × 10^6^) from each group were then added into sterile 96-well cell culture plates. The cell culture supernatant was collected at 24, 72, and 96 h, respectively, after adding the 4 × protein at a final concentration of 10 μg/ml. A mixture of interleukin-2 (IL-2), interleukin-4 (IL-4), interleukin-10 (IL-10), interleukin-12 (IL-12) and interferon-γ (IFN-γ) Capture Beads (BD Biosciences) were added to each sample, incubated in the dark for 2 h, and then washed with 500 μl of washing buffer, followed by centrifugation at 300 × *g* for 5 min. The levels of IL-2, IL-4, IL-10, IL-12 and IFN-γ were measured using flow cytometry (BD Biosciences) employing a BD^™^ Cytometric Bead Array (CBA) kit (BD Biosciences).

### Quantitative real-time reverse transcription PCR (qRT-PCR)

qRT-PCR was carried out to analyze the mRNA levels of cytokines (*n* = 3). Firstly, the total RNA of spleen cells was extracted using the TRIzol reagent (Invitrogen, Waltham, MA, USA) following the manufacturer’s protocol. The cDNA was then synthesized using a First Strand cDNA Synthesis Kit (ReverTra Ace -α-, Toyobo, Osaka, Japan) for use as templates in the qPCR reaction. The qPCR reaction was conducted using a 20 μl mixture comprising 10 μl of 2 × Real time PCR Master Mix (Toyobo), 0.4 μl of each primer (10 µmol/L), 1 μl of cDNA template, and 8.2 μl of sterile distilled water. The primers used in this study are listed in Table S1. Amplification was performed on a CFX96 Touch™ Real-Time PCR Detection System (Bio-Rad, Hercules, CA, USA) and the relative mRNA levels were calculated by the comparative ΔCt method using the formula − 2^−ΔΔCt^ [21].

### Lymphocyte proliferation assay

The splenocyte suspensions from five mice in each group were prepared as described above. The splenocytes (1 × 10^5^) were added to 96-well plates and stimulated with 4 × protein (5 μg/ml) or an equal volume of DMEM high-glucose medium as the negative control. Moreover, wells with no cells but only the medium were used as the blank control. The lymphocytes were cultured in a cell incubator at 37 °C with 5% CO₂ for 96 h. Cell Counting Kit-8 (CCK-8) reagent (Solarbio, Beijing, China) was then added to the wells, and the plates were incubated for another 4 h. Subsequently, the absorbance value at 450 nm was measured using an ELISA microplate reader (BioTek) to analyze lymphocyte proliferation. The calculation formula was as follows: Cell proliferation activity = (OD₄₅₀ of 4x–OD₄₅₀ of Blank)/(OD₄₅₀ of Control–OD₄₅₀ of Blank).

### Detection of parasite loads

Mice (*n* = 5 per group) that were intraperitoneally infected with 100 tachyzoites of the RH strain were sacrificed at 4 dpi to collect the liver, spleen, and lung tissues. Genomic DNA was extracted using a TIANamp Genomic DNA Kit (Tiangen, Beijing, China) according to the manufacturer’s instructions. The genomic DNA of *T*. *gondii* RH strain was extracted and diluted to obtain a concentration gradient (10^2^, 10^1^, 10^–1^, 10^–2^, 10^–3^, 10^–4^, 10^–5^ and 10^–6^ ng/mL) for use in a standard curve through qPCR based on a 529 bp sequence of *T*. *gondii*. The amplification system was as described above and was performed on a CFX96 Touch™ Real-Time PCR Detection System. The Ct value of each sample was used to obtain the concentration of* T*. *gondii* and the parasite burden was ultimately represented as the weight of *T*. *gondii* (fg) per milligram of tissue (mg). The specific formula was as follows: concentration × volume/mass of tissue.

### Hematoxylin–eosin (H&E) staining of the liver, spleen, and lung

Staining with H&E was performed systematically to evaluate histopathological alterations in hepatic, splenic, and pulmonary tissues harvested from RH strain-infected mice (100 tachyzoites, i.p.; *n* = 3 per group) at 4 dpi. The liver, spleen, and lung tissues were removed from mice and soaked in 4% paraformaldehyde (Beyotime) at room temperature for 24 h. Then, the tissues were dehydrated in ethanol in solutions of 70%, 80%, 90%, and 100% for gradient dehydration for 30 min each, then clarified with xylene twice for 30 min each. The clarified tissues were placed in molten paraffin for infiltration, kept in an incubator at about 58 − 60 °C for 1 to 2 h for embedding treatment, and sectioned into thin slices of 4 − 6 µm using a microtome. Subsequently, the sections were stained using H&E. The histopathological changes in the tissue were then observed under a microscope.

### Statistical analyses

In this study, the statistical analyses were performed using GraphPad Prism version 8 (GraphPad Software Inc., La Jolla, CA, USA). The log-rank (Mantel–Cox) test was used to compare the survival curves among different groups. Independent Student’s *t*-tests were conducted to compare results between two groups and one-way analysis of variance (ANOVA) was conducted to compare the results among three or more groups. Continuous variables with normal distribution were presents as mean ± standard deviation (SD); non-normal variables were reported as median (interquartile range). In all analyses, *P* < 0.05 was considered statistically significant.

## Results

### Epitope analysis

The mRNA construct comprised five structural elements: the 5'cap structure, the 5'untranslated region (UTR), the open reading frame (ORF) encoding the 4 × protein, the 3'untranslated region (UTR), and the Poly-A tail. Collectively, these elements contributed to making the mRNA construct more complete and stable (Figure S1A). The potential epitopes of the 4 × protein were predicted by DNASTAR software, including the surface probability, antigenic index, hydrophilic plot, and flexible region (Figure S1B). The hydrophilic amino acids are mainly predicted to be in amino acids 251–286, 709–736, 772–801, 890–916, 926–959, 1066–1106, and 1132–1156. The surface probability regions of the 4 × protein were predicted as amino acids 131–141, 326–336, 381–391, 742–753, 890–903, and 1022–1034. The antigen index regions were predicted as amino acids 419–441, 708–731, 772–804, 889–966, 972–1002, and 1042–1072. The flexible regions were predicted as amino acids 204–286, 742–769, 891–912, 924–943, and 1143–1166. The majority of the regions of the 4 × protein were predicted as hydrophilic and moderately hydrophilic regions. Additionally, the 4 × protein has a more desirable surface probability and antigenicity index than SAG1, suggesting its potential use as an mRNA vaccine against *T. gondii* infection.

### Identification of 4x-mRNA-LNP expression in vitro

**Fig. 1:**
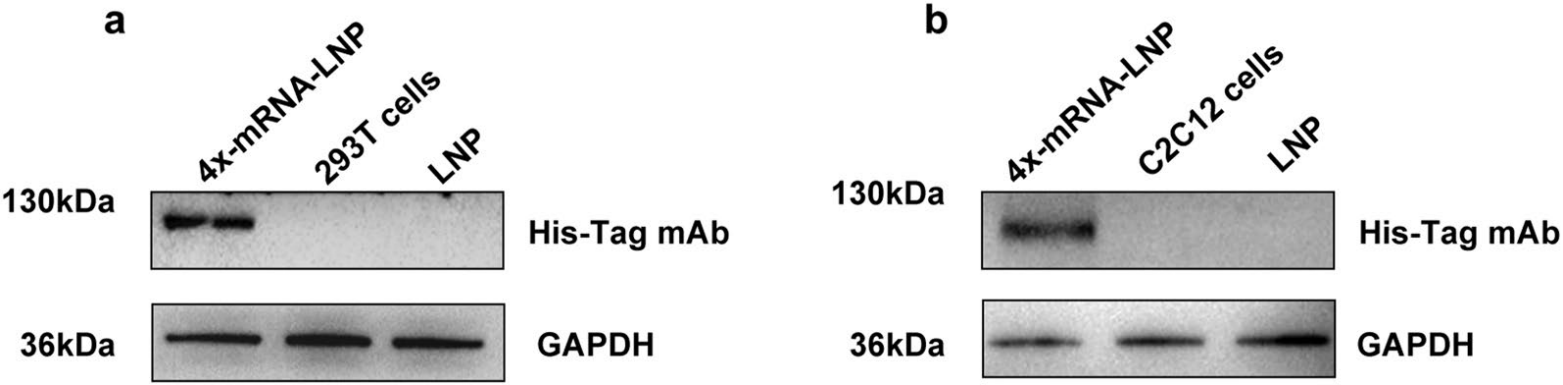
Detection of 4x-mRNA-LNP expression in HEK-293 T cells and C2C12 cells (**a**, **b**). The 4x-mRNA-LNP or LNP were transfected into HEK-293 T or C2C12 cells in 6-well plates using Lip3000 reagent. The HEK-293 T and C2C12 cell lysates from 4x-mRNA-LNP or LNP-transfected or non-transfected cells were collected for western blotting after incubation for 72 h. His-tag mAb-HRP or anti-GAPDH were used as primary antibodies and goat anti-Rabbit-HRP was used as the secondary antibody

The 4x-mRNA-LNP construct was transfected into HEK-293 T cells, and the expression of the 4 × protein was detected using western blotting. As shown in Fig. 1a, a clear single band around 130 kDa was observed in cell samples transfected with 4x-mRNA-LNP when detected using the His-Tag mAb, while no band was detected in samples transfected with LNPs or in untransfected HEK-293 T cells. This suggested that 4x-mRNA-LNP was expressed in the eukaryotic cell line 293 T. Vaccines are frequently administered via intramuscular injection as an immunization route; therefore, it is important to determine whether the vaccine can be correctly expressed in C2C12 (muscle) cells. As shown in Fig. 1b, the results were consistent with those obtained using 293 T cells, proving that 4x-mRNA-LNP could be successfully expressed in C2C12 cells.

### The 4 × recombinant protein was successfully obtained

The 4 × recombinant protein was successfully expressed using the 293 T eukaryotic expression system. Subsequent purification using Ni–NTA (his-tag) affinity chromatography yielded protein fractions that were analyzed using SDS-PAGE. Electrophoretic separation revealed prominent bands within the predicted 100–130 kDa molecular weight range across all elution fractions (25–500 mmol/L imidazole gradient in urea buffer), corresponding to the expected size of the 4 × recombinant protein (Fig. S2a). Immunoblotting with anti-His tag antibodies specifically detected these bands, confirming the successful expression of the target recombinant protein (Fig. S2b).

### Robust humoral and cellular immune responses were elicited by vaccination with the 4x-mRNA-LNP in mice

**Fig. 2:**
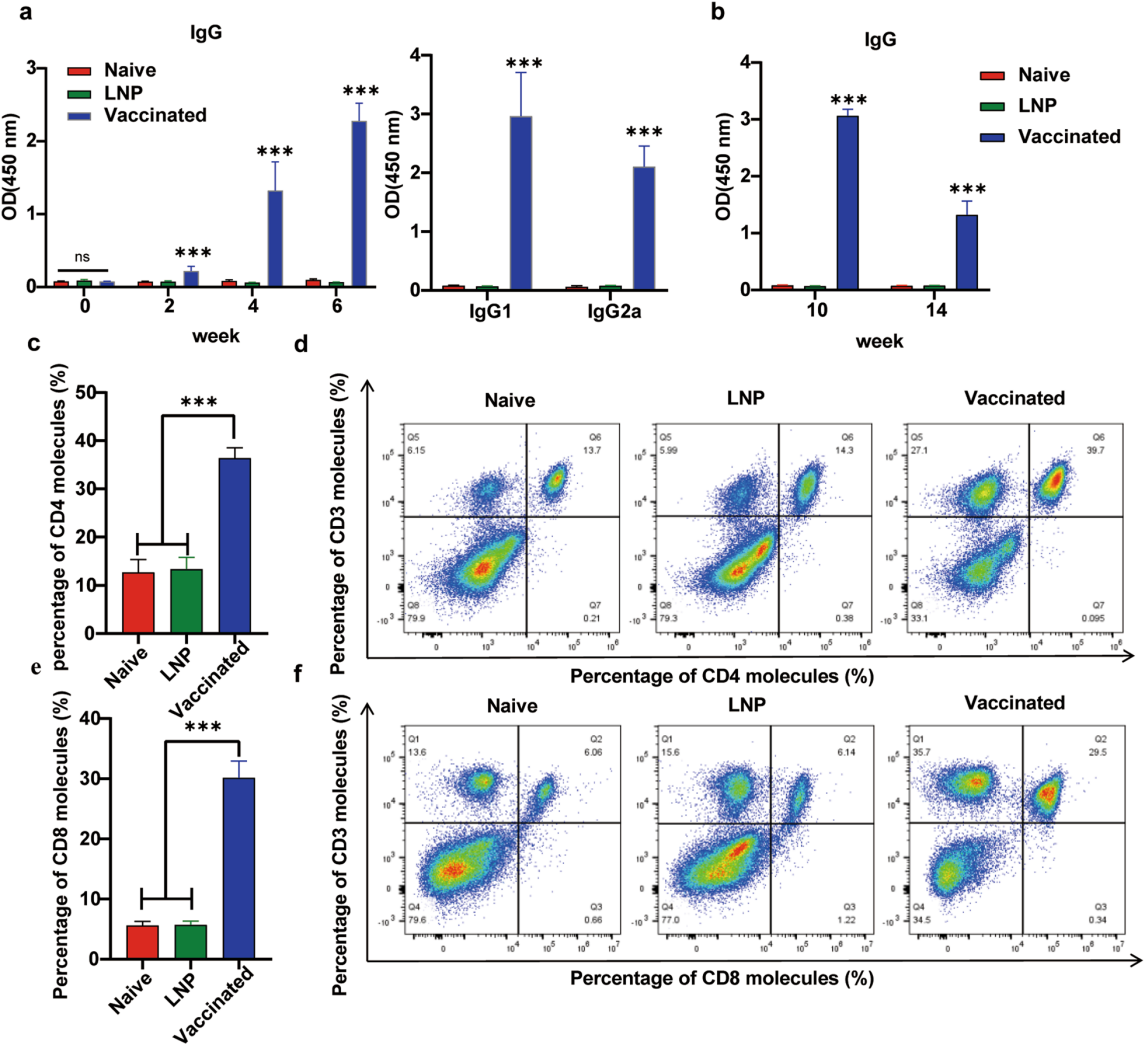
The 4x-mRNALNP vaccine was employed to immunize mice to elicit specific humoral and cellular immunity. **a** The levels of specific IgG antibodies in mouse serum, as assessed using ELISA (coated with 4 × protein) at weeks 0, 2, 4, and 6. Detection of antibody subtypes (IgG1 and IgG2a) in mouse serum at two weeks after the final immunization. **b** The levels of specific IgG antibodies in mouse serum at weeks 10 (6 weeks after the last immunization) and 14 (10 weeks after the last immunization) (*n* = 6), as analyzed using 4 × protein ELISA (**c**-**f**) Detection of CD4^+^ T cell and CD8^+^ T cell populations among peripheral blood mononuclear cells (PBMCs) from immunized BALB/c mice. *n* = 5 per group. Data are presented as the mean ± *SD* of three independent experiments. By one-way ANOVA, *P < 0.05, **P < 0.01, ***P < 0.001, and ns (not significant). *SD* standard deviation

**Fig. 3:**
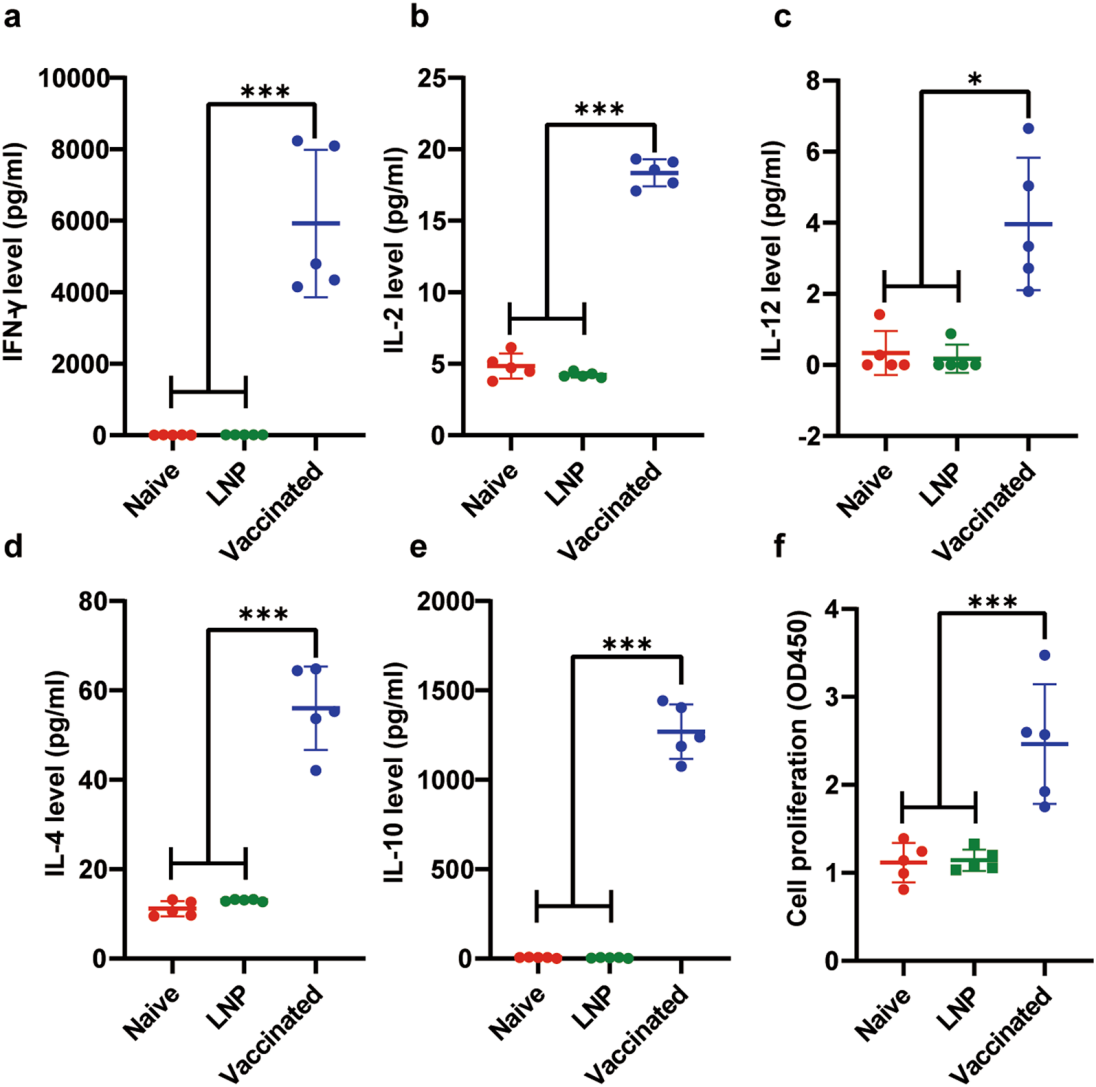
The levels of Th1 and Th2 pro-inflammatory cytokines. Splenocytes collected from mice (*n* = 5) after the final immunization were co-incubated with 4 × protein (5 μg/ml). The levels of Th1 cytokines (IFN-γ (**a**), IL-2 (**b**), and IL-12 (**c**)) and Th2 cytokines (IL-4 (**d**) and IL-10 (**e**)) in the culture supernatant were measured using flow cytometry. **f** The proliferation response of splenocytes from each group was measured using a CCK 8 kit. (*P < 0.05, **P < 0.01, ***P < 0.001), as assessed using by one-way ANOVA

**Fig. 4:**
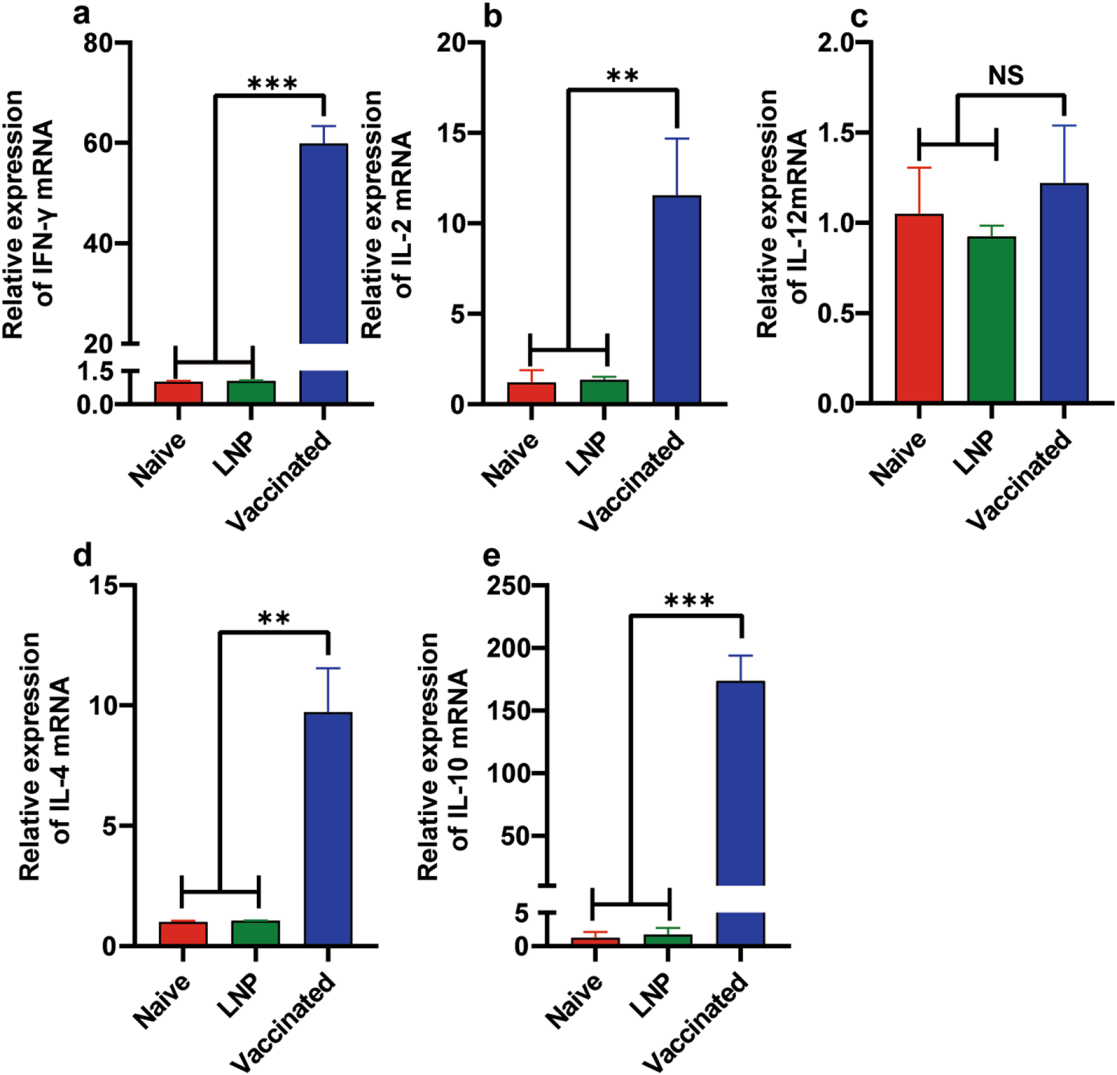
The mRNA expression levels of various cytokines: Th1 cytokines IFN-γ (**a**), IL-2 (**b**), and IL-12 (**c**)), and Th2 cytokines IL-4 (**d**) and IL-10 (**e**) were determined by qRT-PCR. RNA samples were extracted from stimulated splenocytes and reverse transcribed to cDNA for qPCR detection. Each sample was assessed twice (*n* = 5, *P < 0.05, **P < 0.01, ***P < 0.001) using one-way ANOVA

After three immunizations, serum samples collected 2 weeks after each vaccination were analyzed by ELISA using the 4 × recombinant protein as the coating antigen. A notable level of 4x-specific IgG antibodies was detected in the mice vaccinated with 4x-mRNA-LNP compared with those injected with LNP or in naive mice (Fig. 2a). Moreover, the titer of IgG antibodies in the immunized mice increased significantly after each vaccination (*P* < 0.05) (Fig. 2a). Sera samples were collected after the final vaccination to determine the lasting period of immune response. As shown in Fig. 2b, the antibodies could be detected even at 14 weeks (10 weeks after the last immunization), although their levels were decreased compared with those at 10 weeks (6 weeks after the last immunization), suggesting that the mRNA vaccine elicited a strong and long-lasting humoral response. Additionally, the levels of both IgG1 and IgG2a in the immunized mice were markedly higher than those in the control mice (*P* < 0.05) (Fig. 2a) The T cell profile was further determined using flow cytometry. As shown in Fig. 3c–f, the percentages of CD3^+^CD4^+^ T cells and CD3^+^CD8^+^T cells in the 4x-mRNA-LNP vaccinated mice were significantly increased in comparison with those in the control group at two weeks after the last immunization (*P* < 0.05). The levels of Th1-related cytokines (IL-2, IL-12, and IFN-γ) and Th2-related cytokines (IL-4 and IL-10) were all significantly increased compared with those in the control groups (Fig. 3a–e). Notably, the levels of two key cytokines for cellular immunity to clear tachyzoites, IFN-γ (5926 ± 921.6 pg/mL) and IL-10 (1269 ± 68.21 pg/mL), were 736-fold and 253.7-fold higher, respectively, in the immunized mice than in the control groups (Fig. 3a–e), suggesting that immunization of mice with 4x-mRNA-LNP generated a Th1/Th2 mixed and Th1-biased cellular immune response. As shown in Fig. 3f, lymphocytes achieved a notable higher degree of proliferation, indicating an effective cellular immune response induced by stimulation with the 4 × protein. In parallel, RNA was extracted from the splenocytes and reverse transcribed into cDNA for qPCR to detect the mRNA levels of cytokines. The mRNA levels of IL-4, IL-10, IL-2, and IFN-γ were all significantly increased in comparison with the those in the control group (Fig. 4a–e). Both the mRNA and protein levels of IL-12 increased compared with those in the control group. However, statistically, there was a significant difference in the protein level, but not in in the mRNA level. Consistent with the mRNA levels of cytokines, a marked increase in IFN-γ and IL-10 levels were observed in the vaccinated mice (Fig. 4a–e). Taken together, the mRNA vaccine successfully induced a Th1/Th2 mixed and Th1-biased immune response.

### The parasite load and pathological sections in the liver, spleen and lung tissues of mice

**Fig. 5:**
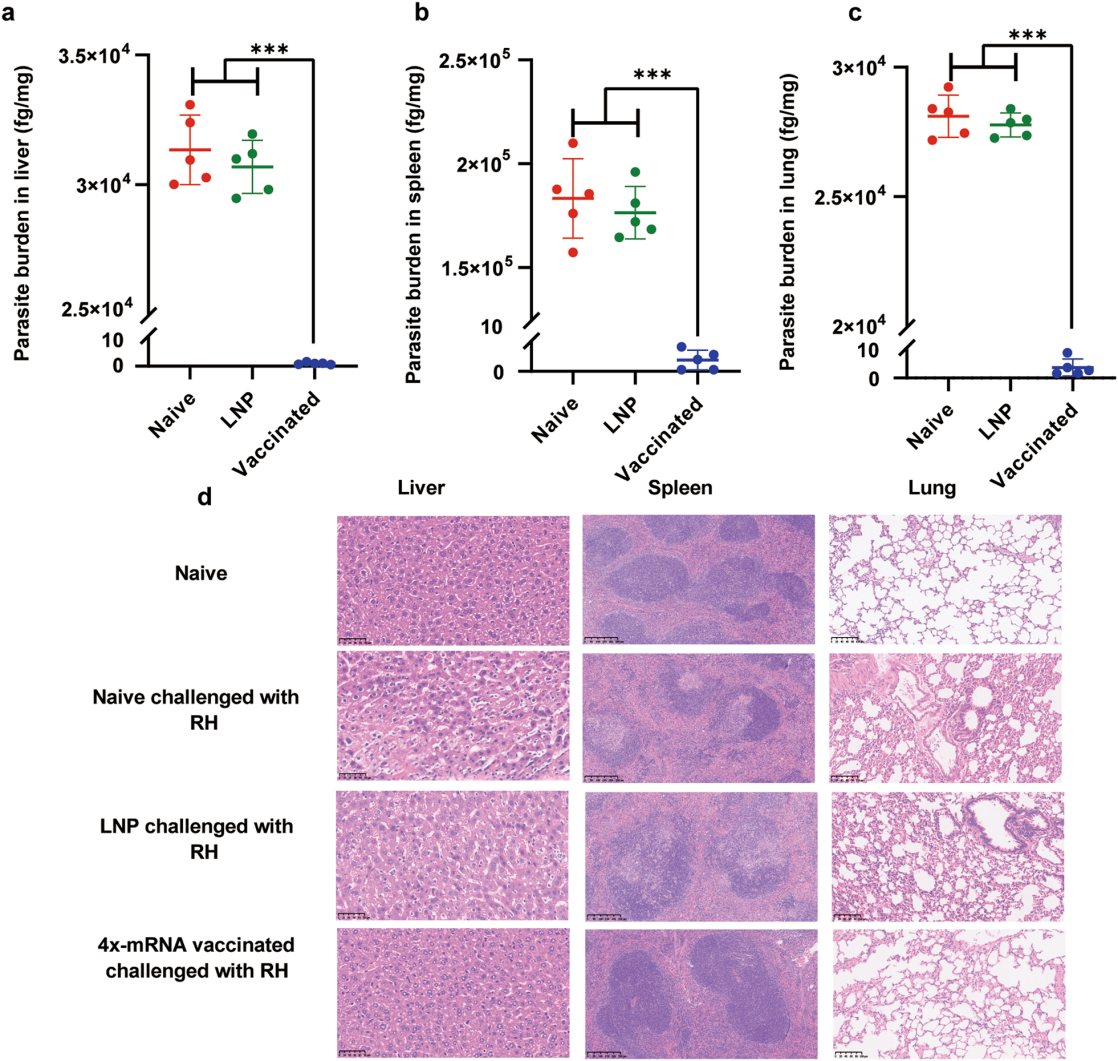
Parasite load in the liver, spleen, and lung of mice infected with the RH strain were detected at 4 dpi by 529 bp based-qPCR assay (**a**–**c**). The protective effect of 4x-mRNA-LNP on mouse tissues: (**d**) Tissue damage in the spleen, liver, and lung following intraperitoneal injection of the RH strain at a dose of 100 at 4 dpi in mice were observed following the final immunization. The results are presented as the mean ± *SD*. (*P < 0.05, **P < 0.01, ***P < 0.001, and ns (not significant). *SD* standard deviation (tested using one-way ANOVA)

Liver, spleen, and lung tissues were collected from all experimental groups at 4 dpi with the RH strain for DNA extraction and subsequent quantification of the parasite load by qPCR. The results demonstrated that tachyzoites were massively replicated in various tissues of the control group. In contrast, fewer than 10 fg of parasites/mg were detected in these tissues from mice injected with 4x-mRNA-LNP (liver: 0.9545 ± 0.3865, spleen: 3.914 ± 3.415, lung: 3.652 ± 2.98) as shown in Fig. 5a–c (*P* < 0.05). H&E staining was employed to evaluate the histopathological changes in RH strain-challenged mice. The results showed that mice immunized with the 4x-mRNA-LNP vaccine exhibited few pathological changes in tissues and organs compared with those in the non-immunized mice. By contrast, tissue samples from *T*. *gondii*-infected mice vaccinated with LNP or non-vaccinated mice displayed obvious pathological changes: a clear cellular separation in liver, the lack of lymphoid follicles with extensively necrotic cells in the spleen, and thicker alveolar walls to varying degrees, indicating severe interstitial pneumonia compared with naive mice (Fig. 5d). Thus, the above results suggested that the 4x-mRNA-LNP vaccine effectively protects against *T. gondii* infection.

### 4x-mRNA-LNP immunization confers protection against infection with various types of *T. gondii* tachyzoites in mice

**Fig. 6:**
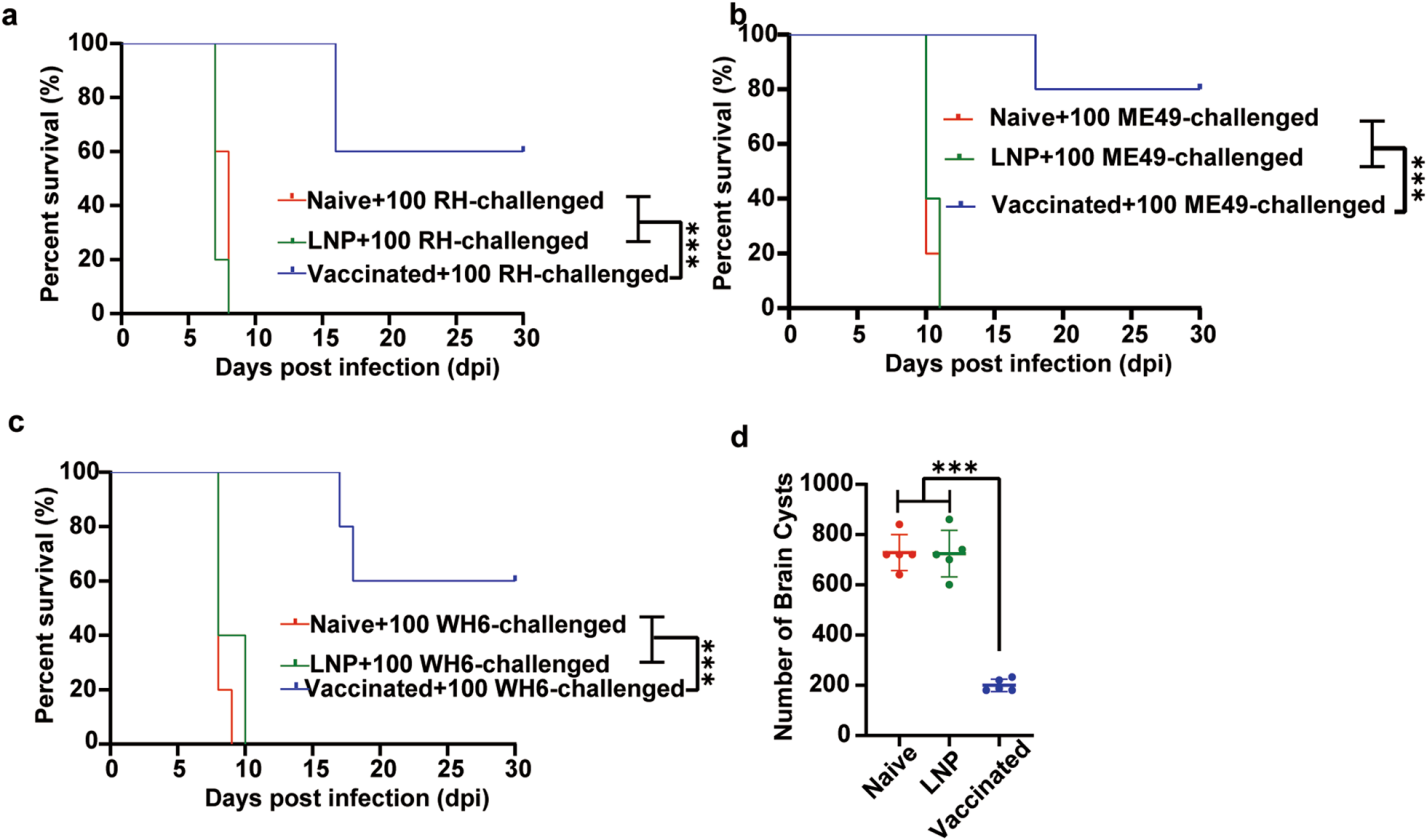
The 4x-mRNA vaccine can induce a protective immune effect against *T. gondii* infection in mice. **a**–**c** Survival curves of mice in each group infected with RH, ME49, and WH6, respectively (5 mice/group); continuously monitored for 30 days. Tested by the log-rank (Mantel–Cox) test, *P < 0.05, **P < 0.01, ***P < 0.001. **d** The cyst loads of the brains in each group infected with 20 oocysts of the type II PRU strain were determined at 40 dpi (5 mice/group). The results are presented as the mean ± *SD*. (*P < 0.05, **P < 0.01, ***P < 0.001, and ns (not significant). *SD* standard deviation (tested using one-way ANOVA)

Two weeks after the final immunization, non-vaccinated and vaccinated mice were challenged with a lethal dose of the type I RH strain. All non-vaccinated mice perished within 8 days. However, only two vaccinated mice died on the 16th day, resulting in a survival rate of 60% within the 30-day observation period (Fig. 6a). Additionally, when challenged with the type II ME 49 tachyzoites or the Chinese local isolate WH6, all the non-vaccinated mice died within 11 and 10 days of infection, respectively. The survival time of mice immunized with 4x-mRNA-LNP was significantly prolonged: 80% and 60% of the mice still survived within the 30-day observation period (Fig. 6b, c). After challenge with 20 oocysts for 40 days, the brain cyst burdens in the immunized mice (200.0 ± 24.94 cysts/brain) were significantly reduced (*P* < 0.05) compared with those in non-immunized mice (728.0 ± 71.55 cysts/brain) (Fig. 6d).

## Discussion

The epidemic protozoan parasite *T. gondii* is the causative agent of the important worldwide zoonosis, toxoplasmosis. The lack of effective and commercially available anti-toxoplasmosis vaccines emphasizes the need for vaccine development to control parasite transmission and prevent the disease [22].

Despite many years of research, the development of anti-toxoplasmosis vaccines remains a significant challenge. The most important reason is the multistage and complex life cycle of *T. gondii*, which results in variations in immunogenicity in response to variations in protein expression levels. Consequently, protective immunity elicited against one particular stage of the parasite might be ineffective when infected by parasites at other stages. Thus, a cocktail vaccine comprising multistage antigens is a highly recommended strategy. Using a reverse vaccinology approach or through proteomic analysis, antigens have been discovered to be effective for vaccine development, such as surface antigens (SAGs), rhoptry proteins (ROPs), microneme proteins (MICs), and dense granule proteins (GRAs). The antigens selected in the present study were designed to combat parasite infections from different stages. ROP18, a critical rhoptry protein of *T. gondii*, plays an essential role in parasite invasion and intracellular survival. This virulence factor phosphorylates host cell proteins to subvert immune defenses and facilitate infection [2]. Another key protein, MIC13, mediates host cell attachment and invasion during *T. gondii* infection. Additionally, bioinformatic analysis further demonstrated that MIC13 expression is significantly upregulated during chronic infection and during in vitro bradyzoite differentiation under stress [14]. Furthermore, ROP18 and MIC13 DNA nucleic acid vaccines both yielded favorable results against chronic and acute infections [15, 16]. *TGME49_237490* and *TGME49_268230* are abundantly expressed at all stages of oocyst formation. These genes might play a crucial role in the reproductive cycle of the parasite and could serve as important targets for further research and the development of novel control strategies against *T. gondii* infection.

Herein, we developed a self-replicating mRNA vaccine (abbreviated as 4x-mRNA) containing four *T. gondii* antigens: ROP18, TGME49_237490, TGME49_268230, and MIC13, which are all key proteins in different life stages. The vaccine was designed to combat acute and chronic toxoplasmosis, and even the infection caused by oocysts from the sexual reproductive stage. We also analyzed the immunogenicity and humoral and cellular immune responses induced by vaccination, and further studied their immune protective effect in a mouse model.

To date, some saRNA vaccines incorporating cocktail therapy have been developed in field of infectious diseases and cancer [23], such as HPV and SARS-CoV-2 saRNA vaccines [24, 25]. However, until now, there has been no report of a multi-gene combined self-replicating mRNA vaccine in the field of *T. gondii*.

Our results and analysis demonstrated that the designed tetravaccine displayed remarkable immunogenicity and protective efficacy against *T. gondii* infection. The tetravaccine was able to stimulate both humoral and cellular immune responses. It induced the long-term production of specific antibodies, including IgG and its subclasses, which play crucial roles in neutralizing the parasites and activating complement pathways. Additionally, the tetravaccine promoted the activation and proliferation of T cells, particularly CD4^+^ and CD8^+^ T lymphocytes. These T cells are capable of secreting cytokines and performing effector functions. The enhanced immune responses conferred by the tetravaccine resulted in a significant reduction in parasite loads and improved survival rates in challenged animals. These characteristics make the tetravaccine a promising candidate for a vaccine against toxoplasmosis.

Long-term immune protection after immunization is important. In most studies, *T. gondii* challenges were often performed two weeks after the last vaccine dose, with a few examples performed at 8 weeks [26]. Therefore, how long the immunity generated by the vaccine will last is a pivotal feature. Here, we used a self-replicating mRNA system for vaccine construction, which has several advantages. The self-replicating mRNA used in this study carries the RdRp sequence, which, once expressed, could be used as a template to produce more cRNA, thereby significantly increasing the duration of mRNA in the cells and enhancing the immune response. We discovered that the specific IgG antibodies could be determined at least 10 weeks after the last vaccination, indicating that the 4x-mRNA induced long-term humoral immunity. In addition, mRNA vaccines do not necessitate nuclear entry, thereby precluding the risk of integration into the host genome, implying that mRNA vaccines are safer compared with DNA vaccines.

In this work, very high specific IgG levels, as well as those of its subtypes (IgG1 and IgG2a), were achieved, especially after three injections of the 4x-mRNA-LNP vaccine. The production of specific antibodies is an important parameter to measure immune protection, because anti-*T. gondii* antibodies can suppress its invasion into host cells and cause immune-induced cytotoxic effects through neutralizing effects or antibody-mediated activation of the classical complement pathway [27, 28]. As a Th1 response marker, IgG2a antibodies play a crucial role in fighting against *T. gondii* infection. Thus, given the high protection levels in challenged mice, it was reasonable to observe a noticeable increase in IgG2a titers.

The nature of *T. gondii* means that the immune cells that combat infection are cytotoxic T lymphocytes (CTLs) and helper T cells of the Th1 subset, which engage in cellular immunity and cytokine secretion, especially IFN-γ. Thus, the assessment of these immune parameters is crucial to determine the immunogenic potential of a vaccine.

During *T. gondii* invasion, T-cell-mediated immunity is the dominant immune response that mediates resistance to parasite infection. To be specific, upon activation, CD4^+^ T lymphocytes are capable of activating macrophages, secreting cytokines, and migrating to sites of infection, thus contributing to the immune response during the initial stage of *T. gondii* infection [29]. Concurrently, activated CD8^+^ T cells can undergo differentiation into cytotoxic T lymphocytes [30], which can mediate cell lysis [31]. We found significantly higher levels of CD3^+^ CD4^+^ T cells and elevation of CD3^+^ CD8^+^ T cells in mice immunized with 4x-mRNA-LNP compared with those in the control group. In addition, we also found that the lymphocyte proliferative activity increased significantly when stimulated by the 4x-antigen, indicating an effective immunotoxicity against *T. gondii*.

It should be emphasized that the importance of cytokines in clearing the intracellular parasite, typically as indicators of Th1 (IFN-γ, IL-2 and IL-12) and Th2 (IL-4, IL-10) responses, is crucial. IL-12 can effectively stimulate the production of IFN-γ and decrease the parasite load at parasite-infected sites [32, 33]. IL-2, as a key cytokine for T-cell activation, can stimulate the proliferation and differentiation of CD4^+^ T and CD8^+^ T cells, and induce T cells and natural killer (NK) cells to secrete cytokines (including IFN-γ), thereby playing a crucial role in the resistance against *T. gondii* infection. Moreover, IFN-γ can induce a variety of intracellular mechanisms to kill parasites or inhibit their replication and proliferation [34–37]. Thus, it is necessary to assess these cytokines to confirm the polarization of the immune response triggered by 4x-mRNA-LNP. In our experiment, a significantly higher level of IL-2 and a slightly higher level of IL-12 were detected in vaccinated mice compared with those in the control. At the same time, a markedly higher level of IFN-γ was observed, which could be explained by the presence of a large population of activated T cells. As mentioned above, IFN-γ plays a crucial role in *T. gondii* eradication, thus a high level of IFN-γ is desirable to provide protection against attack by a lethal dose of the parasite.

However, an overly high level of Th1 inflammatory factors might cause pathological damage to mice and even lead to death [38]. Thus, a balance between Th1 and Th2 responses is needed for a favorable outcome for mice [39, 40]. Consistently, we observed that the expression levels of IL-4 and IL-10 were significantly elevated upon stimulation with 4 × antigens, especially IL-10, which was approximately 1000-fold higher than that in the control group. IL-10 is able to protect the body from severe tissue damage induced by *T. gondii* infection by suppressing the inflammatory response and modulating immune functions [41]. Regarding IL-4, it can facilitate B cell activation and the generation of antibodies, particularly antibody subtypes such as IgE and IgG1 [42]. Furthermore, these antibodies play a role in the resistance against *T. gondii* infection via antibody-dependent cell-mediated cytotoxicity (ADCC). Thus, the notably increased levels of IL-10 and IL-4 in this work could be beneficial to prevent excessive Th1-type responses from causing pathological damage to mice and in combating toxoplasmosis.

In most anti-toxoplasmosis vaccine studies, Th2 cytokines were significantly induced, while a few studies observed no statistically significant production of IL-10, which could be explained if the aim was to achieve a better Th1 response [43]. Together, the results show that in mice, 4x-mRNA-LNP can induce a mixed Th1 and Th2 immune response, with a Th1 bias, and plays a significant role in the process of resisting *T. gondii* infection.

The major aim of this work was to discover whether our 4x-mRNA-LNP vaccine could provide protection against toxoplasmosis in immunized mice. Except for a slight inflammation in the lung, we found few histopathological changes and remarkable decreases in parasite loads in the liver, spleen, and lung of mice immunized with 4x-mRNA-LNP and infected with the RH strain. In addition, 60% of vaccinated mice survived during the 30-day observation period, indicating robust protection against acute toxoplasmosis triggered by the RH strain.

As previously described, strain variation in immunogenicity has a high impact and poses a significant challenge for vaccine development. For example, the type I RH strain no longer matches the native strain, which might lead to insufficient cross-protection provide by a single strain. Interestingly, we observed comprehensive protective effects against both the type II ME49 strain and the Chinese clinical isolated strain WH6. WH6 exhibits lower replication rates in host cells compared with the RH strain and demonstrated intermediate virulence (between RH and PRU) [44] in dose-dependent challenge experiments, with survival rates ranging from 60 to 80%. Although it does not induce complete protection, the extended survival times induced by 4x-mRNA-LNP were somewhat satisfactory compared with certain single-antigen mRNA vaccines [45–47].

Another important observation is that the mice vaccinated with 4x-mRNA-LNP showed a significant decrease (72.5%) in the number of brain cysts when challenged with oocysts of the PRU strain, compared with that in the control mice. Furthermore, the reduction in brain cyst load induced by this four-gene cocktail saRNA vaccine was greater than the 62.1% reduction achieved by RREP-NTPase-II/LNP, single antigen saRNA vaccine [47].

To sum up, our results suggested that a cocktail mRNA vaccine is a promising approach to control *T. gondii* infection compared with single-antigen-based vaccination. It should be stressed that our 4x-mRNA-LNP, containing two critical antigens required for the development of oocysts, was designed to confer protection against different life stages and various parasite strains. Therefore, this result strongly suggests broad immunity protection against oocysts, as well as tachyzoites and cysts. Besides, our results indicated that the 4x-mRNA-LNP vaccine might contribute to controlling toxoplasmosis in cats, the definitive host of *T. gondii*, thereby reducing oocyst production and release. However, this proposition undoubtedly needs further evaluation using an optimal immune procedure in cats.

It should be noted that this study did not evaluate potential antigenic interference or synergistic effects among the four components, which could significantly impact immune outcomes. Additionally, although the vaccine demonstrated promising efficacy, comprehensive toxicological assessments, including long-term effects and dose-dependent safety profiles, should be further investigated in higher mammals.

## Conclusions

Our study shows that 4x-mRNA-LNP, containing *T. gondii* proteins ROP18, TGME49_237490, TGME49_268230 and MIC13, administered with LNPs, stimulated a robust immune response. This resulted in effective long-term immunity, as demonstrated by the observation of histopathological changes, the assessment of protection rates, and a decrease in parasite loads or the cyst burden. Of note, our results indicated that 4x-mRNA-LNP is a promising vaccine candidate, given that the elicited immunity is protective against various strains and different infectious forms of *T. gondii*. Furthermore, the 4x-mRNA-LNP induced long-term anti-toxoplasmosis immunity, which is a significant factor in determining the vaccine's utility, and indicates its sustained protection against infection. 

## Supplementary Information

Additional file 1.

Additional file 2.

Additional file 3.

Additional file 4: Figure S1. The composition of the quadrivalent mRNA vaccine and the prediction of protein epitopes.The main structural elements of 4x-mRNA.Comparative analysis of linear B cell epitope characteristics between the 4x protein and SAG1, including hydrophilicity, flexible regions, antigenic index, and surface probability, as predicted using DNASTAR.

Additional file 5: Figure S2. Purification and characterization of the 4x recombinant protein.SDS-PAGE analysis of the 4x recombinant protein eluted with urea-imidazole buffer at increasing concentrations.Western blotting verification of the recombinant protein's immunogenicity using an anti-His tag monoclonal antibody conjugated with horseradish peroxidaseas the primary antibody.

Additional file 6: Table S1. qRT-PCR primers designed to amplify IL-2, IL-4, IL-10, IL-12, IFN-γ, and Gapdh genes.

## Data Availability

The datasets used and/or analyzed during the current study are available from the corresponding author upon reasonable request.

## Abbreviations

4x-mRNA-LNP: Self-replicating mRNA vaccine consisting of four antigens of *T. gondii*: ROP18, TGME49_237490, TGME49_268230 and MIC13
HEK-293 T cells: Human embryonic kidney 293 T cells
C2C12 cells: Mouse myoblast cells
RdRp: RNA-dependent RNA polymerase
FACS: PBS with 0.5% FBS
DMEM: Dulbecco’s Modified Eagle medium
FBS: Fetal bovine serum
PBS: Phosphate buffered saline
ELISA: Enzyme-linked immunosorbent assay
TMB: 3,3’,5,5’-Tetramethylbenzidine
CBA: Cytometric bead array
CCK-8: Cell counting kit 8
*SD*: Standard deviation
