# Supplementary material for: A novel combined quadrivalent self-amplifying mRNA-LNP vaccine provokes protective immunity against acute and chronic toxoplasmosis in mice
Source: Infect Dis Poverty. 2025 Jun 23;14:55. doi: 10.1186/s40249-025-01332-6 (PMC12183821; doi:10.1186/s40249-025-01332-6)
Supplement: Supplementary file 1 — Additional file 1. [file 40249_2025_1332_MOESM1_ESM.docx]

**The original amino acid sequence**

**ROP18 (348-554)**

**MFSVQRPPLTRTVVRMGLATLLPKTACLAVLNVALVFLLFQVQDGTGITLDPSKLDSKPTSLDSQQHVADKRWPATVGHYKYLAGATESTRDVSLLEERAQHRVNAQETNQRRTIFQRLLNLLRRRERDGEVSGSAADSSSRPRLSVRQRLAQLWRKAKSFFTRGIPRYFSQGRNRLRSLRAQRRRSELFFEKADSGCVIGKRILAHMQEQIGQPQALGNSERLDRILTVAAWPPDVPERFVSVTTGETRTLVRGAPLGSGGFATVYEATDVETNEELAVKVFMSEKEPTDETMRDLQRESFCYRNFSLAKTAKDAQERCRFMVPSDVVMLEGQPASTEVVIGLTTRMVPNYFLLMMRAETDMSKVISWVFGDASVNNSELGLVVRMYLSSQAIRLVANVQAQGIVHTDIKPANFLLLKDGRLFLGDFGTYRINNSVGPAIGTPGYEPPERPFQTTDITYTFTTDAWQLGITLYCIWCKERPTPADGIWDYLHFADCPSTPELVQDLIRNLLNREPQKRMLPLQALETAAFNEMDSVVKRAAQNFEQQEHLHTE**

**TGME49_237490 (41-251)**

**MATVGNPGSPSHGGPGVSSPVGGNRPSAVRATSGMGNETGAKGKCRSHGGAGAKSRRSVRVELSEEQRKDIKEAFDLFDTEGTGSIDAKELKVALRALGFEPTKEEMKKLLNEIEKKRREPDVMKMISTSNPNAAAAVAASAPVAQTATTSLGQLDFNEFLEILTIKINEKPTREQISRGKALRGNPEGWAGFRMLAGPTGVIGWKEMKKAAVELGEKLTDEELREMLNHASHSHNKGVVTEEDFLRILRA**

**TGME49_268230 (41-316)**

**MRASPVRGLNSSAPGRASTPSQCGACASSAPTACFLAPPSCLPFFFSCTFLLRIWCISIDLCLPTVEPSPRWNREPLCVVTFASCPSTVSRCPQVEVEPVSVVCADGSAPSASFADAGEQPFMRCLREEFEPSVALCSEGFDLHAATSRCVTTVKEAPGWTCPTGYRLPEHELPAQSAKEVVAPHLGSRKGDSPSPVVVGPPLPRVQAGFCERLEYAEVQFVCPVGFGREKDKKAKEWVCVAQKAVPATHLCDSGFFLEGDACVMHLTMAPSLVDQDGRPFPCVTRGDGSNSCGHAADWQGSLGVERETAKKHGKR**

**MIC13 (48-468)**

**MVGVFQLCLLTCLLCLLKGSDAIQLRQHRNGLGVEQSFVSEETDIVGGRISSLQAELDSYCHNRYKQLCDGGNKKFCDKTGVARYGTGIQSQQTAEWRCYFAEVLKPSGRKVQCVDDCGNYFPCLGVVDPNDTFHATAHNQILNFIKDGVEKHCSPFQMAGNNYCQGVLADTVARKDTGTASQKAKAWRCYKKDSLTYEARSICVDNCGGEIECPGGRSETSGELLSQHYTREKELQKVIESQSAPCHDACVATPINPPICADTESLLQSTKRSRAQRFIDDACQKLFNCHCKKDKKFCTTVVARKDRGRAGSQNDAEWRCYSLEELDFQKTNPSCIDECGNELPCQGAVPEESTHHITWTKLPSKIDEATQKFCTERQRAANDYCASEFADSLARYGPGAGDQTSQFRCIKKAAMRADAEGQCADSCKGTETCGGGRSRLDGPLSVDDVLNAHEGISEAMKSASTEC**

The blue color represents the sequence after truncation according to the antigenic epitope.

**The amino acid sequence after 4x optimization**

**MDAMKRGLCCVLLLCGAVFVSPMVPNYFLLMMRAETDMSKVISWVFGDASVNNSELGLVVRMYLSSQAIRLVANVQAQGIVHTDIKPANFLLLKDGRLFLGDFGTYRINNSVGPAIGTPGYEPPERPFQTTDITYTFTTDAWQLGITLYCIWCKERPTPADGIWDYLHFADCPSTPELVQDLIRNLLNREPQKRMLPLQALETAAFNEMDSVVKRAAQNFEQQEHLHTEGSAGSAAGSGEFAKGKCRSHGGAGAKSRRSVRVELSEEQRKDIKEAFDLFDTEGTGSIDAKELKVALRALGFEPTKEEMKKLLNEIEKKRREPDVMKMISTSNPNAAAAVAASAPVAQTATTSLGQLDFNEFLEILTIKINEKPTREQISRGKALRGNPEGWAGFRMLAGPTGVIGWKEMKKAAVELGEKLTDEELREMLNHASHSHNKGVVTEEDFLRILRAGSAGSAAGSGEFCLPFFFSCTFLLRIWCISIDLCLPTVEPSPRWNREPLCVVTFASCPSTVSRCPQVEVEPVSVVCADGSAPSASFADAGEQPFMRCLREEFEPSVALCSEGFDLHAATSRCVTTVKEAPGWTCPTGYRLPEHELPAQSAKEVVAPHLGSRKGDSPSPVVVGPPLPRVQAGFCERLEYAEVQFVCPVGFGREKDKKAKEWVCVAQKAVPATHLCDSGFFLEGDACVMHLTMAPSLVDQDGRPFPCVTRGDGSNSCGHAADWQGSLGVERETAKKHGKRGSAGSAAGSGEFGRISSLQAELDSYCHNRYKQLCDGGNKKFCDKTGVARYGTGIQSQQTAEWRCYFAEVLKPSGRKVQCVDDCGNYFPCLGVVDPNDTFHATAHNQILNFIKDGVEKHCSPFQMAGNNYCQGVLADTVARKDTGTASQKAKAWRCYKKDSLTYEARSICVDNCGGEIECPGGRSETSGELLSQHYTREKELQKVIESQSAPCHDACVATPINPPICADTESLLQSTKRSRAQRFIDDACQKLFNCHCKKDKKFCTTVVARKDRGRAGSQNDAEWRCYSLEELDFQKTNPSCIDECGNELPCQGAVPEESTHHITWTKLPSKIDEATQKFCTERQRAANDYCASEFADSLARYGPGAGDQTSQFRCIKKAAMRADAEGQCADSCKGTETCGGGRSRLDGPLSVDDVLNAHEGISEAMKSASTECHHHHHH**

**The nucleotide sequence after 4x optimization**

**ATGGACGCCATGAAGAGGGGCCTGTGCTGCGTGCTGCTGCTGTGTGGCGCCGTGTTCGTGAGCCCCATGGTACCAAACTATTTTCTTCTCATGATGCGGGCAGAAACGGACATGAGCAAAGTCATTTCATGGGTATTTGGAGATGCGTCTGTCAATAACAGTGAATTAGGCCTGGTCGTTCGAATGTACCTATCCAGTCAGGCAATCAGACTAGTGGCCAATGTTCAAGCTCAGGGAATTGTGCATACGGATATCAAACCGGCGAATTTCCTCCTCTTGAAAGACGGTCGCCTGTTTCTCGGCGACTTCGGAACGTATAGAATCAATAATTCGGTTGGACCCGCGATAGGTACTCCCGGTTACGAGCCTCCGGAGCGACCGTTTCAGACTACAGACATCACCTATACATTCACCACTGACGCGTGGCAACTCGGTATAACTTTGTACTGCATCTGGTGCAAGGAACGTCCAACTCCGGCCGACGGCATCTGGGACTACTTACACTTCGCAGATTGTCCTTCCACGCCTGAGCTGGTTCAAGACCTCATCCGAAACCTCTTGAATCGAGAGCCTCAGAAACGGATGCTCCCGCTACAAGCCTTGGAGACCGCAGCGTTTAACGAGATGGATTCAGTAGTAAAACGCGCCGCGCAAAACTTCGAACAGCAGGAACATCTCCACACAGAAgggtcagctggctccgctgctggttctggcgagtttGCAAAGGGAAAATGCCGGAGTCACGGCGGAGCTGGGGCGAAGAGCCGTCGAAGCGTTCGCGTGGAACTCAGCGAAGAGCAGCGGAAAGACATCAAAGAGGCATTCGATCTGTTCGACACGGAAGGAACAGGCAGCATCGACGCGAAAGAGTTGAAGGTCGCTCTGCGGGCTCTCGGGTTCGAGCCCACAAAGGAAGAAATGAAGAAGCTGCTGAACGAGATCGAGAAGAAACGCCGCGAACCAGACGTCATGAAAATGATTTCCACCAGCAACCCGAACGCAGCAGCCGCCGTCGCAGCCTCAGCGCCAGTTGCGCAGACCGCAACGACGTCCCTGGGGCAGTTGGACTTCAACGAGTTTCTTGAGATTCTCACAATCAAAATCAACGAGAAGCCGACAAGGGAACAAATCTCTCGAGGCAAGGCACTTCGAGGAAACCCGGAGGGGTGGGCGGGCTTCCGCATGTTAGCTGGTCCTACGGGCGTCATCGGTTGGAAAGAGATGAAGAAAGCTGCGGTTGAACTCGGAGAGAAACTTACAGACGAAGAACTGCGCGAGATGCTCAACCACGCGTCTCACTCACACAATAAGGGGGTCGTGACCGAAGAAGACTTTCTTCGCATCCTTCGAGCTgggtcagctggctccgctgctggttctggcgagtttTGTCTGCCGTTCTTCTTTTCATGCACTTTCCTTCTCCGCATTTGGTGCATCTCCATCGACTTGTGTCTGCCTACCGTCGAGCCGTCCCCCAGATGGAACCGCGAACCCCTCTGCGTTGTGACATTCGCCTCCTGTCCGTCCACCGTGTCGCGTTGCCCCCAGGTGGAGGTGGAGCCGGTCTCCGTGGTCTGCGCCGACGGCTCTGCGCCTTCTGCCTCCTTTGCGGACGCGGGAGAGCAGCCGTTCATGCGTTGCCTGCGCGAAGAGTTCGAGCCGAGTGTCGCGCTCTGCAGCGAAGGCTTTGATCTACACGCGGCAACAAGCCGGTGCGTGACGACGGTGAAGGAGGCCCCTGGGTGGACTTGTCCAACGGGATATCGTTTACCTGAGCACGAGTTGCCGGCCCAGAGCGCGAAAGAGGTCGTCGCTCCACACCTCGGCTCGCGAAAAGGAGACAGTCCGTCCCCCGTCGTCGTCGGCCCGCCACTGCCCCGCGTCCAGGCCGGCTTCTGCGAGCGTCTGGAGTACGCCGAAGTCCAGTTCGTCTGTCCTGTTGGATTCGGAAGAGAAAAAGACAAGAAAGCCAAGGAGTGGGTATGCGTCGCCCAGAAAGCCGTCCCCGCCACGCACCTTTGCGACTCGGGATTCTTCCTTGAAGGCGACGCATGCGTCATGCATTTGACGATGGCGCCCTCTCTCGTGGACCAAGACGGCAGGCCATTCCCCTGCGTAACTCGAGGCGACGGCTCCAACTCGTGTGGGCATGCAGCCGACTGGCAGGGCTCTTTGGGCGTGGAAAGGGAGACTGCGAAGAAGCATGGAAAGCGGgggtcagctggctccgctgctggttctggcgagtttGGACGCATTTCTTCCCTGCAAGCCGAGTTGGACTCCTACTGCCACAATCGGTACAAGCAACTCTGCGACGGCGGCAACAAAAAATTCTGTGACAAAACTGGTGTCGCTCGCTACGGCACGGGGATCCAGAGCCAGCAGACTGCCGAATGGAGATGCTACTTTGCGGAGGTTTTGAAACCCTCAGGCCGCAAGGTGCAGTGTGTCGATGACTGCGGGAATTATTTCCCCTGTTTGGGAGTTGTGGATCCGAACGACACTTTCCACGCGACTGCACATAATCAGATTTTGAATTTCATCAAGGATGGAGTTGAAAAGCACTGCAGTCCATTCCAAATGGCTGGCAACAATTATTGCCAGGGCGTTCTTGCGGACACAGTCGCCCGCAAAGACACGGGCACCGCATCTCAAAAGGCGAAAGCGTGGCGCTGCTATAAGAAAGACAGTCTCACGTACGAGGCTAGATCCATTTGCGTGGACAACTGCGGCGGTGAAATAGAATGCCCTGGCGGACGTTCAGAAACCTCTGGAGAACTACTCAGTCAGCATTATACTAGGGAAAAAGAGCTGCAGAAAGTGATCGAGTCACAAAGTGCCCCCTGCCATGACGCATGCGTTGCGACACCCATCAACCCACCAATTTGCGCCGACACTGAGAGTCTCCTGCAAAGTACCAAACGCAGCAGAGCACAGAGATTCATCGATGACGCGTGCCAGAAACTGTTCAACTGCCACTGCAAAAAAGACAAGAAATTCTGCACGACTGTAGTCGCCCGCAAGGATCGAGGTCGCGCAGGCAGTCAAAATGATGCTGAGTGGCGCTGCTATTCTTTAGAAGAGTTGGACTTTCAAAAAACAAACCCGTCGTGCATTGATGAGTGCGGAAATGAACTGCCGTGCCAAGGCGCAGTACCAGAAGAGTCTACTCACCACATAACGTGGACCAAGTTGCCGTCTAAAATTGACGAAGCTACGCAAAAGTTCTGCACTGAACGACAAAGAGCTGCGAATGACTATTGCGCGTCAGAGTTCGCAGACAGTCTGGCGCGATATGGACCCGGCGCGGGGGACCAAACCAGTCAATTTCGGTGTATTAAAAAGGCGGCAATGCGTGCAGATGCCGAAGGGCAATGTGCCGATTCCTGCAAAGGAACGGAAACGTGCGGAGGCGGTCGAAGCAGACTTGATGGCCCTCTCTCAGTTGATGACGTTTTGAATGCTCACGAGGGTATTTCAGAGGCTATGAAAAGCGCCTCGACAGAGTGCCACCACCACCACCACCAC**

The red color represents the signal peptide. The green color represents ROP18, TGME49_237490, TGME49_268230, and MIC13 respectively. The black color represents the His-Tag. The blue represents the flexible fragment.
