# Supplementary figures and images for: A novel combined quadrivalent self-amplifying mRNA-LNP vaccine provokes protective immunity against acute and chronic toxoplasmosis in mice
Source: Infect Dis Poverty. 2025 Jun 23;14:55. doi: 10.1186/s40249-025-01332-6 (PMC12183821; doi:10.1186/s40249-025-01332-6)

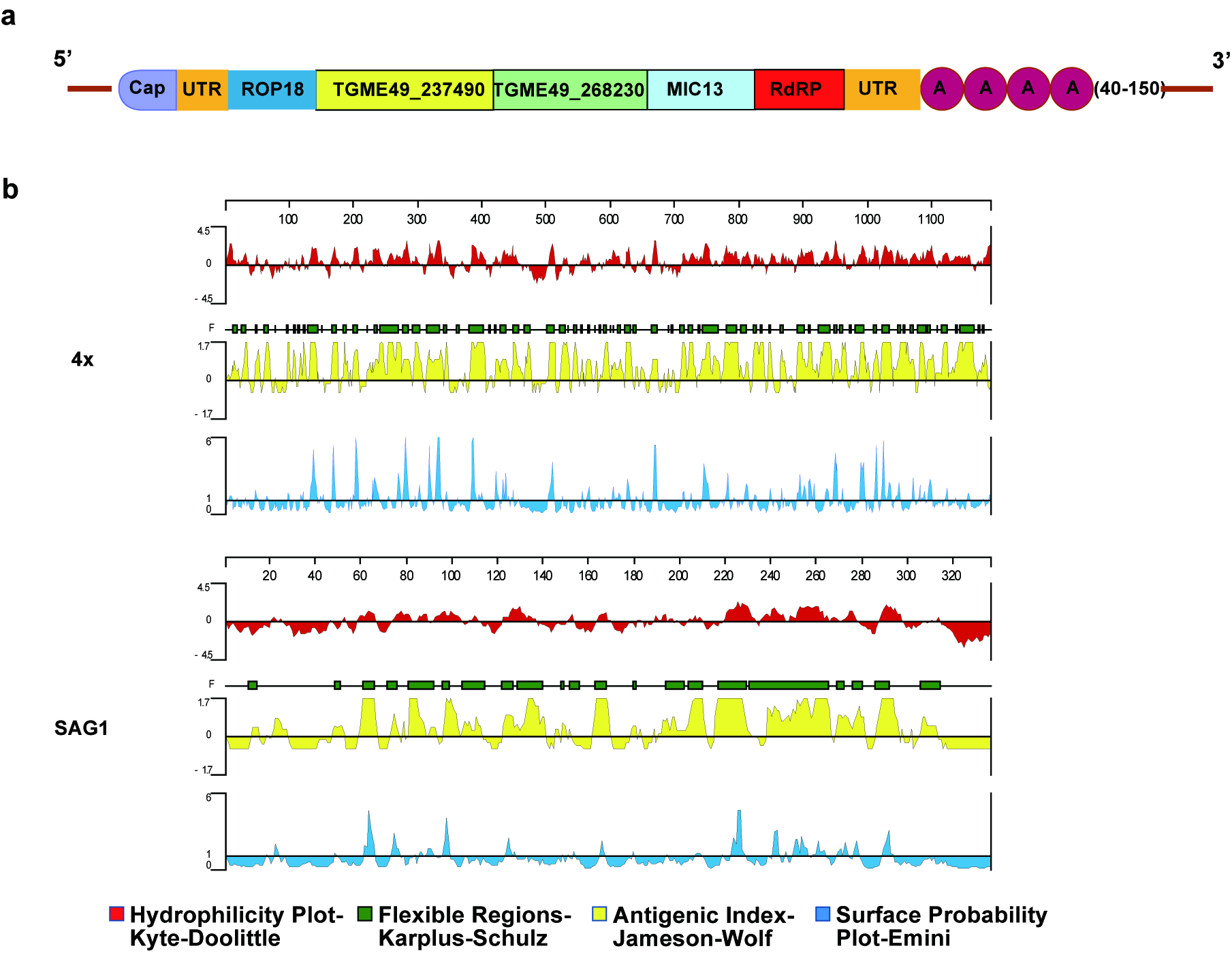

Supplement: Supplementary file 4 — Additional file 4: Figure S1. The composition of the quadrivalent mRNA vaccine and the prediction of protein epitopes.The main structural elements of 4x-mRNA.Comparative analysis of linear B cell epitope characteristics between the 4x protein and SAG1, including hydrophilicity, flexible regions, antigenic index, and surface probability, as predicted using DNASTAR. [file 40249_2025_1332_MOESM4_ESM.tif]

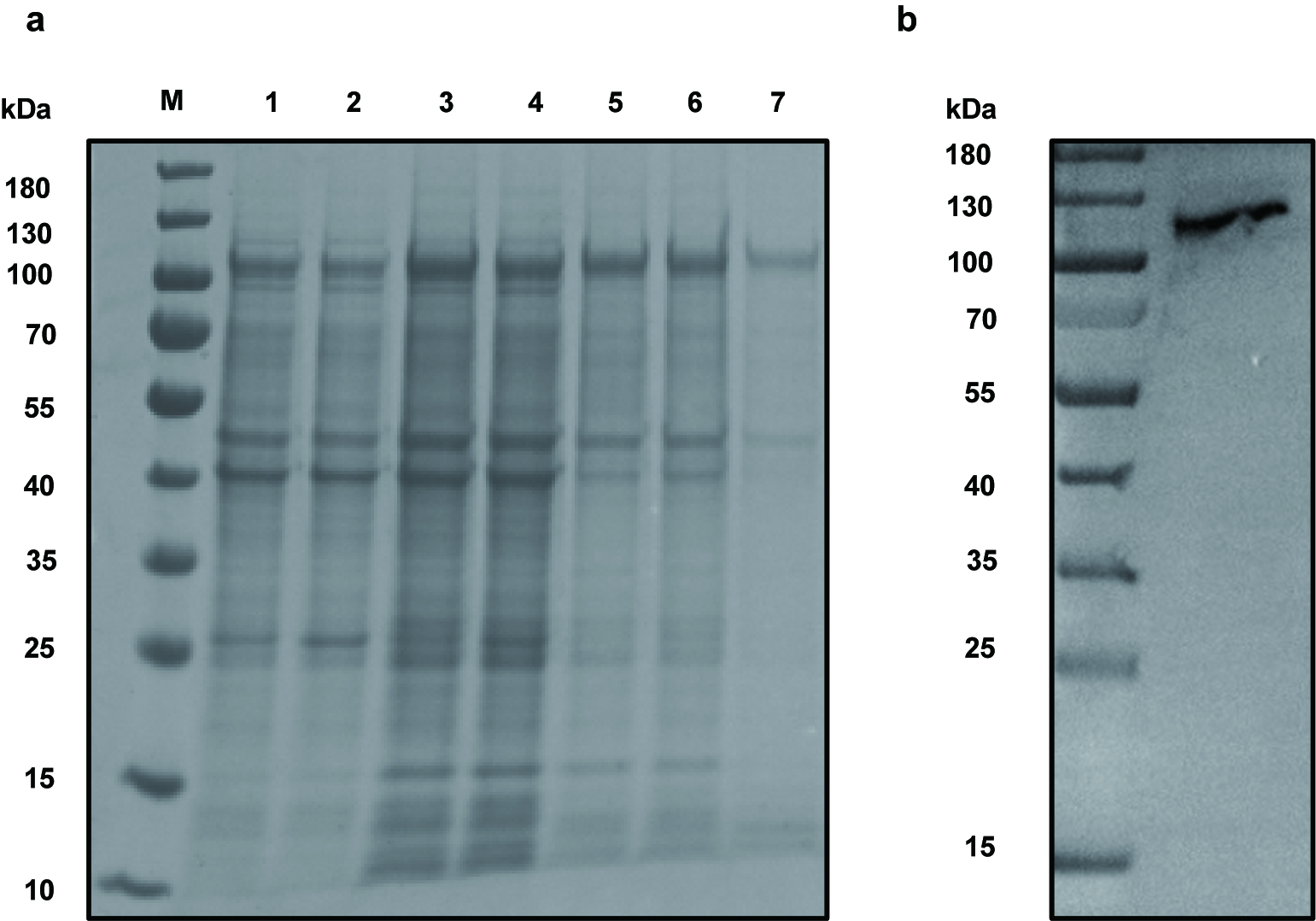

Supplement: Supplementary file 5 — Additional file 5: Figure S2. Purification and characterization of the 4x recombinant protein.SDS-PAGE analysis of the 4x recombinant protein eluted with urea-imidazole buffer at increasing concentrations.Western blotting verification of the recombinant protein's immunogenicity using an anti-His tag monoclonal antibody conjugated with horseradish peroxidaseas the primary antibody. [file 40249_2025_1332_MOESM5_ESM.tif]
