# Supplementary material for: A novel combined quadrivalent self-amplifying mRNA-LNP vaccine provokes protective immunity against acute and chronic toxoplasmosis in mice
Source: Infect Dis Poverty. 2025 Jun 23;14:55. doi: 10.1186/s40249-025-01332-6 (PMC12183821; doi:10.1186/s40249-025-01332-6)
Supplement: Supplementary file 6 — Additional file 6: Table S1. qRT-PCR primers designed to amplify IL-2, IL-4, IL-10, IL-12, IFN-γ, and Gapdh genes. [file 40249_2025_1332_MOESM6_ESM.docx]

| **Primer name** | **Sequence** |
| --- | --- |
| IL-2-F | 5'-TGAGCAGGATGGAGAATTACAGG-3' |
| IL-2-R | 5'-GTCCAAGTTCATCTTCTAGGCAC-3' |
| IL-4-F | 5'-TCAACCCCCAGCTAGTTGTC-3' |
| IL-4-R | 5'-TCTGTGGTGTTCTTCGTTGC-3' |
| IL-10-F | 5'-GCTCCTAGAGCTGCGGACT-3' |
| IL-10-R | 5'-TGTTGTCCAGCTGGTCCTTT-3' |
| IL-12-F | 5'-GATGTCACCTGCCCAACTG-3' |
| IL-12-R | 5'-TGGTTTGATGATGTCCCTGA-3' |
| IFN-γ-F | 5'-ATGAACGCTACACACTGCATC-3' |
| IFN-γ-R | 5'-CCATCCTTTTGCCAGTTCCTC-3' |
| GAPDH-F | 5'-CAGTGGCAAAGTGGAGATTG-3' |
| GAPDH-R | 5'-TGCCGTGAGTGGAGTCATAC-3' |
